# Inhibition of Inducible Nitric Oxide Synthase (iNOS) by Andrographolide and *In Vitro* Evaluation of Its Antiproliferative and Proapoptotic Effects on Cervical Cancer

**DOI:** 10.1155/2021/6692628

**Published:** 2021-03-16

**Authors:** Akbar Pasha, Divya Vishambhar Kumbhakar, Ravinder Doneti, Kiran Kumar, Gangappa Dharmapuri, Pavan Kumar Poleboyina, Heena S. K., Preethi Basavaraju, Deepthi Pasumarthi, Annapurna S. D., Pavani Soujanya, I. Arnold Emeson, Vijayalaxmi Bodiga, Smita C. Pawar

**Affiliations:** ^1^Department of Genetics & Biotechnology, University College of Science, Osmania University, Hyderabad, 500 007 Telangana, India; ^2^Department of Bioinformatics, School of Biosciences & Technology, Vellore Institute of Technology, Vellore, Tamil Nadu 632014, India; ^3^Department of Animal Biology, School of Life Sciences, University of Hyderabad, Hyderabad, 500 046 Telangana, India; ^4^Department of Pathology, Osmania Medical College, Hyderabad, 500095 Telangana, India; ^5^Department of Human Genetics and Molecular Biology, Bharathiar University, Coimbatore, 641046 Tamil Nadu, India; ^6^Institute of Genetics and Hospital for Genetic Diseases, Osmania University, Begumpet, Hyderabad, 500007 Telangana, India

## Abstract

This work is aimed at investigating the expression levels of inducible nitric oxide synthase (iNOS) in cervical cancer and identifying a potential iNOS inhibitor. The data mining studies performed advocated iNOS to be a promising biomarker for cancer prognosis, as it is highly overexpressed in several malignant cancers. The elevated iNOS was found to be associated with poor survival and increased tumor aggressiveness in cervical cancer. Immunohistochemical and RT-PCR investigations of iNOS showed significant upregulation of endogenous iNOS expression in the cervical tumor samples, thus making iNOS a potent target for decreasing tumor inflammation and aggressiveness. Andrographolide, a plant-derived diterpenoid lactone, is widely reported to be effective against infections and inflammation, causing no adverse side effects on humans. In the current study, we investigated the effect of andrographolide on the prognostic value of iNOS expression in cervical cancer, which has not been reported previously. The binding efficacy of andrographolide was analyzed by performing molecular docking and molecular dynamic simulations. Multiple parameters were used to analyze the simulation trajectory, like root mean square deviation (RMSD), torsional degree of freedom, protein-root mean square fluctuations (P-RMSF), ligand RMSF, total number of intramolecular hydrogen bonds, secondary structure elements (SSE) of the protein, and protein complex with the time-dependent functions of MDS. Ligand-protein interactions revealed binding efficacy of andrographolide with tryptophan amino acid of iNOS protein. Cancer cell proliferation, cell migration, cell cycle analysis, and apoptosis-mediated cell death were assessed *in vitro*, post iNOS inhibition induced by andrographolide treatment (demonstrated by Western blot). *Results*. Andrographolide exhibited cytotoxicity by inhibiting the *in vitro* proliferation of cervical cancer cells and also abrogated the cancer cell migration. A significant increase in apoptosis was observed with increasing andrographolide concentration, and it also induced cell cycle arrest at G1-S phase transition. Our results substantiate that andrographolide significantly inhibits iNOS expression and exhibits antiproliferative and proapoptotic effects on cervical cancer cells.

## 1. Introduction

Cervical cancer (CC) ironically is still the foremost common prevalent cancer in women globally, though being the most preventable disease. The incidence of cervical cancer is on an increase, with a decrease in the age of onset [1]. Cervical cancer is most commonly caused by the infection of high-risk HPV infection (HPV16/18); the integration of the HPV genetic material into the host genome intrudes the immune response and increases the inflammation, thus promoting cancer progression [2]. The HPV infection remains inert and takes 10-20 years to develop into cancer. Persistent HPV infection alone is not sufficient to develop cervical cancer; various molecular actions and physiological factors play a vital role in the progression of the disease [3]. Smoldering inflammatory response, oxidative stress, and epigenetic changes caused by persistent HPV infection play a critical role in development of cancer [4]. The cells like macrophages, dendritic cells, and mast cells adjacent to the cancer cells create the tumor microenvironment. Inflammation is a response of the immune defense mechanism to harmful stimuli released by various infected cells; it releases cytokines, ROS, and hormones to maintain this inflammatory response. This persistent inflammation plays a significant role in carcinogenesis and contributes to cellular transformation, proliferation, invasion, and angiogenesis [5]. Inflammatory molecules involved in inflammation-mediated cervical cancer consists of reactive oxygen species (ROS), inducible nitric oxide synthase (iNOS), TNF-*α*, interleukin-1, IL-6, IL-8, IL-18, hypoxia-inducible factor (HIF), cyclooxygenase-2 (COX-2), matrix metalloproteinase enzyme-9 (MMP-9), and chemokines [6, 7]. Inflammation and oxidative stress are two conditions assisted by persistent HPV infection leading to carcinogenesis. Chronic inflammation impairs cell homeostasis and leads to oxidative stress thus increasing the production of reactive oxygen and nitrogen species (RONS), which in turn modulates the receptor-mediated signaling pathway, transcriptional activation for the cellular proliferation, differentiation, and death signaling as second messengers in signal transduction [8, 9]. The nitric oxide synthases are a family of isoenzymes which convert L-arginine to L-citrulline and generate NO species. Commonly, cells possess three isoforms of NOS: inducible (iNOS), endothelial (eNOS), and neural (nNOS). nNOS and eNOS are constitutively expressed in neurons and endothelial cells, respectively. Inducible NOS is expressed in macrophages, neutrophils, endothelial cells, hepatocytes, and many other cancer cell types. The NOS2 expression is triggered by cytokines and can lead to local accumulation of high concentrations of NO for extended stages [10]. The abnormal concentrations of NO implicate pathogenicity and carcinogenicity [11]. Cellular high concentration levels of NO are associated with enhanced vascular permeability facilitating tumor growth by enhanced blood flow [12]. Inducible nitric oxide synthase (iNOS/NOS2) isoform is calcium-dependent and highly expressed in many cancers. NOS2 is the only isoform to maintain the levels of NO and playing both procarcinogenic and anticarcinogenic roles in different cancers [13–15]. Various cancers known for enhanced iNOS levels are related to bacterial or viral infection like cervical, gastric, prostate, and esophageal cancers and hepatocellular carcinoma [16–18]. iNOS expression correlates with VEGF and together is very vital for tumor growth; high levels of NO produced by the NOS2 triggers the angiogenesis process in the tumors by regulating the VEGF expression. NOS2 knockdown in SiHa and HeLa cells has shown a decrease in cell proliferation due to the decrease in the concentration of VEGF thus confirming that the NOS2 regulates the growth of cervical cancer cells in a VEGF-dependent process [14]; thus, effective inhibition of iNOS expression will thereby reduce the NO levels, triggered by inflammatory stimuli in cancer cells, and hence pronounces to be a valuable therapeutic strategy. Natural products as alternative therapeutics for treating various diseases are getting more attention in the ever-emerging medical field. Bioactive compounds from medicinal plants are known for offering relief from symptoms of various diseases. Antioxidant, anti-inflammatory, antiaging, antiatherosclerosis, and anticancerous plant-derived compounds such as polyphenols, tocopherols, ascorbic acid, carotenoids, and flavonoids with their free radical scavenging activity and physiological activities have been used extensively as therapeutics in various diseases [19–21]. Andrographolide is a bicyclic diterpenoid lactone isolated from leaves of *Andrographis paniculata*, a Chinese herbal medicine used as an anti-inflammatory drug for the treatment of bacterial infections, laryngitis, diarrhea, rheumatoid arthritis, and inflammatory diseases. Andrographolide has shown potent effect on rats suffering endotoxaemia by preventing the NO production through inhibiting iNOS [22–24]. In our current study, we establish that andrographolide exerts its anti-inflammatory effect by inhibiting the iNOS, thus reducing the NO production and curtailing the source for ROS and RNS. The aim of this study was to elucidate the interaction of andrographolide with iNOS by employing *in silico* molecular docking and molecular dynamic simulations and evaluate its inhibitory activity on the iNOS in cervical cancer HeLa cells.

## 2. Materials and Methods

### 2.1. Clinical Sample Analysis

#### 2.1.1. Sample Collection

For the study, 40 women subjects were recruited, which included 30 cancer patients suffering from cervical lesions and 10 normal controls within a range of 30-50 yrs of age. Invasive cervical cancer patients were clinically tested for cancer diagnosis at the oncology department in MNJ Cancer Hospital, Hyderabad, India. Age-matched control subjects seeking health care with no individual history of cervical cancer were randomly procured from the department of gynecology, CC Shroff Hospital, Hyderabad, India. The study was approved by the ethical committee of MNJ Cancer Hospital, Hyderabad, India, and written consent was obtained from both the patients and control subjects involved in the study.

#### 2.1.2. Immunohistochemical Staining (IHC)

For IHC staining, paraffin-embedded specimens of both control and cervical cancer tissue were cut at a thickness of 4 mm using a microtome and eventually deparaffinized and rehydrated followed by PBS washing. The antigen was revived in citrate buffer (0.01 M) for 15 mins. The sections of the specimen were then blocked with endogenous peroxidase for 10 mins at 37°C using H_2_O_2_ (3%) in methanol and further blocked with goat serum (10%, 10 mins). The sections were incubated with a primary anti-iNOS antibody (Abcam, USA) at dilution of 1 : 200 and kept overnight at 4°C. Post incubation, the sections were washed with PBS and were incubated with a biotin-labeled secondary antibody for 15 mins at 37°C, followed by treatment with streptavidin peroxidase reagent for 10 mins. The specimen was then subjected to 4 min incubation with 3,3′-diaminobenzidine (DAB containing 0.0018% H_2_O_2_ in 0.05 M Tris-HCl buffer: pH 7.6) solution, and the specimen sections were then counterstained using hematoxylin and eosin for visualization. Slides were then washed with distilled water and air-dried. The specimen slides were permanently mounted using a coverslip. IHC staining of iNOS on tissue was assessed following reported protocols [25]. The specimen sections were then observed under an inverted light microscope (Magnus INVI Olympus, Noida, India) at 4x and 10x magnification. The specimen staining was scored with intensity in the range of 0-5% taken as 0, i.e., negative; 6–25% taken as 1, i.e., weak; 26–50% taken as 2, i.e., medium; and 51–100% taken as 3, i.e., strong. The score was determined by adding the intensity of staining with its extent.

#### 2.1.3. Quantitative RT-PCR Analysis

Total RNA was isolated from tissue samples (cervical biopsies and healthy controls) using a Qiagen RNA isolation mini kit (Qiagen, Cat No: 73404) as per the manufacturer's protocol. Gene primers and amplicon size are represented in Table 1. The quality and quantity of isolated RNA were assessed using a biospectrophotometer (Eppendorf). Total RNA (1 *μ*g/*μ*l) from both tumor and nontumor samples were reverse-transcribed using a PrimeScript™ 1^st^ strand cDNA synthesis kit (Cat# 6110A), from which 1 *μ*l of reverse-transcribed RNA was used to determine the expression of iNOS by using SYBR Green Master Mix (SYBR® Premix Ex Taq™ II Universal, Cat# RR82LR) in real-time PCR (Agilent AriaMx detection system), with a final volume of 20 *μ*l reaction in each well. The protocol consists of 40 cycles at 95°C for 3 min (hot start), 95°C for 0.05 seconds (melting), and 52°C for 30 sec (annealing) and extension at 72°C for 20 sec. All the samples were processed in triplicates, and the beta-actin gene was used as an endogenous control for reference. The results were normalized with endogenous control. Statistical analysis for qPCR was performed using GraphPad Prism 6 (GraphPad Software Inc., San Diego, CA), and the mean ± standard deviation (SMD) of all the values was calculated by the Student *t*-test with a significant *p* value < 0.05.

### 2.2. *In Silico* Analysis

#### 2.2.1. Data Mining of NOS2 Gene

Gene Expression Profiling Interactive Analysis (GEPIA: http://gepia.cancer-pku.cn/) is an interactive web application for RNA sequencing expression. We investigated the box plots of NOS2 gene expression in different cancers by comparing cancer and normal tissue from the TCGA (The Cancer Genome Atlas) datasets [26].

#### 2.2.2. Molecular Docking Studies

*(1) Ligand Structure Preparation*. Molecular docking was applied to explore the mechanism of ligand binding activity and to correlate the binding score of the ligand. In our present study, we have selected natural compound andrographolide as ligand based on biological activity. The 3D structure of the phytochemical andrographolide was obtained from PubChem https://pubchem.ncbi.nlm.nih.gov/ with ID: 5318517. Docking has been carried out by AutoDock Vina. The ligand was minimized, and the least confirmation *E* = 535.66 was picked up and converted to pdbqt format.

*(2) Protein Preparation for Docking*. The 3D structure of the protein iNOS was downloaded from the database Protein Data Bank (PDB) (http://www.rcsb.org/structure/4nos) (4NOS) which is the PDB ID of the protein target. The protein pdb structure was loaded to PyRx (Vina) 0.8 and converted to macromolecules, where pdbqt format of the protein is generated that includes Kollman charges. Water was removed, and polar hydrogen was added to the protein.

*(3) Molecular Dynamic Simulations*. Molecular dynamic (MD) simulation was accomplished to determine the interactions between andrographolide (ligand) and nitric oxide synthase (iNOS) with PDB ID: 4NOS in an aqueous environment. Molecular dynamics is one of the most persuasive methods to study the physical nature and crucial properties of molecules such as their interaction, diffusion, and stability concerning each other. A molecular dynamic study was adopted to examine the structural dynamics and stability of the native nitric oxide iNOS (4NOS) and iNOS-andrographolide complex. By using the Glide module of the Schrodinger software, the binding affinity of iNOS-andrographolide was derived as -7.8 kcal/mol. In our present analysis, binding affinities of andrographolide and protein 4NOS were investigated by conducting static molecular docking. Molecular dynamic simulation of the andrographolide and 4NOS was performed after energy minimization of the system [27]. The OPLS force field was used for all the energy minimization and MD. Molecular dynamic trajectories were analyzed to understand the conformational changes of the native protein caused due to identified interactions between andrographolide and 4NOS. We carried out the 30 nanoseconds of molecular dynamic simulations for the protein iNOS in the native state and the iNOS complex with the ligand. Multiple parameters were used to analyze throughout the simulation trajectory, like root mean square deviation (RMSD), torsional degree of freedom, protein-root mean square fluctuations (P-RMSF), ligand RMSF, total number of intramolecular hydrogen bonds, secondary structure elements (SSE) of the protein, and protein complex with the time-dependent functions of MDS [28]. All the molecular dynamic simulations were performed with a CPU Dell Precision T5810 Intel Xeon V3 processor with 10 cores and 20 threads 240 GBs SSD 32 GBs DDR4 RAM GPU NVIDIA GeForce RTX 2060 super 8GBs DDR6 RAM 256-bit bus size.

### 2.3. *In Vitro* Analysis

#### 2.3.1. Cell Culture

Cervical carcinoma HeLa cells and normal human embryonic kidney (HEK) 293 cells were seeded and maintained in DMEM with 10% FBS and 1% penicillin-streptomycin. Both the cells were cultured in a humidified incubator with 5% CO_2_ at 37°C.

#### 2.3.2. MTT Assay for Cell Viability Assessment

Andrographolide was procured from Sigma Aldrich Lot # MKCF4812 (St. Louis, MO, U.S.A.) and was solubilized in 100 mM dimethylsulphoxide (DMSO) and further diluted with PBS. Effect of andrographolide on cell proliferation was estimated using the 3-(4,5-dimethylthiazole-2-yl)-2,5-biphenyl tetrazolium bromide (MTT) (Promega, Madison, WI, USA) assay. The HeLa and HEK cells were cultured to a density of 3 × 10^3^ cells/well in 96-well plates and incubated at 37°C in 5% CO_2_ overnight. Fresh DMEM containing an increasing concentration of andrographolide (0, 5, 10, 20, 40, and 80 *μ*M) was added to the wells of both HeLa and HEK cells. After 24 h incubation, 20 *μ*l of MTT (5 mg/ml in PBS) was added to each well and incubated for 4 h at 37°C. The plates were incubated with 100 *μ*l of DMSO for 30 min in a shaker incubator. The optical density was measured at 590 nm to estimate the extent of MTT reduction to formazan using a microplate reader (Bio-Rad). Three separate independent experiments were carried out, and data is represented as the mean value of triplicates. The cell viability percentage was calculated with respect to untreated cells taken as the control for each cell line. The concentration essential for 50% of growth inhibition (IC_50_) was calculated using GraphPad Prism software.

#### 2.3.3. Western Blotting

HeLa cells treated with andrographolide (2, 4, 8, and 10 *μ*M) and the untreated HeLa cells (taken as control) were grown in 6-well plates for 24 hrs, later harvested and washed with prechilled PBS. The cells were then resuspended and lysed with radioimmunoprecipitation assay (RIPA) buffer (containing phosphorylase and protease inhibitor) for 60 min at 4°C. The cells were centrifuged at 12,000 rpm for 15 min at 4°C. The concentration of protein was determined using the Bradford assay. An equal quantity of 25 *μ*g protein lysate was loaded on SDS-PAGE and then electrotransferred onto a nitrocellulose membrane (0.2 *μ*M Bio-Rad cat # 1620112). The membranes were then blocked using 5% skimmed fat-free milk and incubated for 60 min. The membrane was then blotted with NOS2 (1 : 1000 dilution) and GAPDH (Santa Cruz Biotechnology SC-25778) followed by overnight incubation at 4°C. The membranes were washed (2 × 15 min) with TBST (Tris-buffered saline having 0.1% Tween-20, pH 7.5) and incubated with a horseradish peroxidase-conjugated secondary antibody (Santa Cruz Biotechnology SC-2004 and SC-2005 at 1 : 5000 dilution) for 1 h at room temperature and then washed with TBST multiple times. The antibody-bound protein blots were visualized by chemiluminescence (Bio-Rad ChemiDoc MP imaging™ system). The experiments were performed in three biological triplicates.

#### 2.3.4. DAPI (4′,6-Diamidino-2-phenylindole)

The changes in nuclear cell morphology and chromatin structure are due to andrographolide treatment-induced apoptosis; this was analyzed using fluorescent DNA binding dye DAPI (4′,6-diamidino-2-phenylindole). The HeLa cells were treated with the andrographolide doses of 5 and 10 *μ*M based on 50% inhibitory concentration (IC_50_) and incubated for 24 h at 37°C with 5% CO_2_. Untreated HeLa cells were the control. The cells were fixed with 3.8% paraformaldehyde and incubated with DAPI (0.5 *μ*g/ml in PBS) in the dark (15 min at 37°C) followed by PBS washes. The apoptotic cells with condensed and fragmented nuclei were viewed under an inverted fluorescence microscope (EVOS™ M5000, CA, USA).

#### 2.3.5. *In Vitro* Scratch Wound Healing Assay

The effect of andrographolide on the migration of HeLa cells was assessed using a scratch wound healing assay. The cells were seeded on 6-well plates and incubated at 37°C, with 5% CO_2_ for 24 h. The cells were grown to 90% confluence, and the monolayer of cells was scraped uniformly with a sterile p10 micropipette tip and washed with PBS to remove scraped detached cells. The cells were treated with fresh medium containing 5 and 10 *μ*M andrographolide and incubated at 37°C for 24 h. Cell migration into the wound of both treated and untreated HeLa cells (control) was measured at 0 h and 24 h. The photomicrographs of migrated cells in all cases were captured (at 4x magnification) using an inverted fluorescence microscope (EVOS™ M5000, CA, USA), and the scratched area was calculated with ImageJ software (version 1.50i, National Institute of Health, Bethesda, MD, USA).

#### 2.3.6. Cell Cycle Analysis

The effect of andrographolide on the DNA of HeLa cells was analyzed using PI dye (BD Biosciences, Mountain View, CA, USA) for cell cycle analysis with BD FACSVerse™ (Becton Dickinson, USA). HeLa cells were grown to a density of 2 × 10^5^ cells per well in a 6-well microplate, followed by treatment with 5 and 10 *μ*M andrographolide, for 24 h (5% CO_2_, 37°C). Post incubation, the cells were collected by centrifugation and fixed with 70% chilled ethanol at −20°C then washed with PBS and resuspended in ice-cold PBS (0.5 ml). The cells were stained with PI in RNase A solution (50 *μ*g/ml PI and 100 *μ*g/ml RNase A) and incubated for 30 min at 4°C. The cell cycle analysis of both treated and untreated (control) cells was analyzed by FACSuite™ software.

#### 2.3.7. Flow Cytometric Analysis of Apoptosis

The translocation of phosphatidylserine from the inner cell membrane to the outer cell membrane is an indicator of apoptosis [29]. Apoptosis induced by andrographolide treatment was quantitatively and qualitatively evaluated using Annexin V-FITC/propidium iodide (PI) double staining (BD Biosciences, Sparks, MD, USA). It determines and distinguishes the cells undergoing necrosis and early and late apoptosis. HeLa cells (3 × 10^4^) were seeded in 6-well plates and treated with 5 and 10 *μ*M of andrographolide for 24 h, and the untreated cells were taken as the control. Cells were harvested using trypsin and centrifuged (1000 g) and fixed with chilled 70% ethanol overnight at 4°C. Cell pellets were washed twice with ice-cold PBS and resuspended in 500 *μ*l of 1x binding buffer. The cells were stained with Annexin V/FITC solution (5 *μ*l) and PI (10 *μ*l of 50 *μ*g/ml) in the dark, at room temperature for 30 min followed by the addition of 400 *μ*l binding buffer (1x), and the cells were analyzed within 60 min of staining. Cells undergoing apoptosis were monitored and measured with BD FACSVerse™ (Becton Dickinson, USA), and data was analyzed using FACSuite™ software.

#### 2.3.8. Statistical Analysis

The data represent three independent experiments for all assessments and are represented as the mean ± standard deviation (SD). One-way ANOVA followed by Tukey's test was used to evaluate the statistical differences, and the statistical analyses were accomplished with GraphPad Prism (version 6.01). *p* values at <0.05 were considered to be statistically significant.

## 3. Results

### 3.1. Immunoexpression of iNOS by IHC

**Figure 1:**
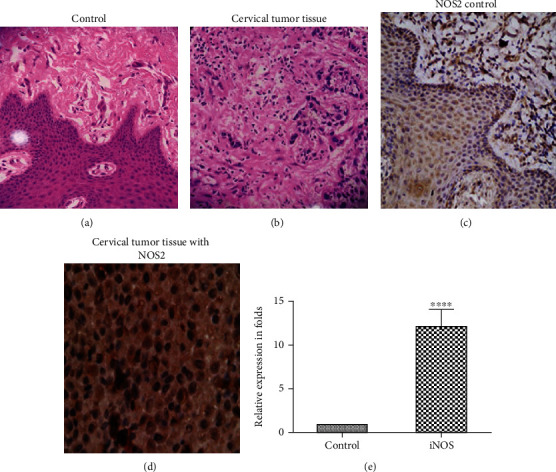
(a, b) H&E (hematoxylin and eosin) staining of (a) control tissue and (b) cervical tumor tissues; (c, d) iNOS antibody-specific tissue staining: (c) control cervix section showing weak iNOS expression and (d) cervical carcinoma section with an elevated level of iNOS expression showing strong intensity. (e) Significantly elevated iNOS expression in cervical cancer tissue in contrast to normal control assessed by RT-PCR.

The expression of iNOS using a monoclonal iNOS antibody via immunohistochemistry (IHC) staining was evaluated in 5 cervical tumor tissues. Photomicrographs revealed H&E staining and immunopositivity of iNOS of cervical cancer tissue which exhibited deformed cellular morphology and irregular and deeply stained nuclei (Figure 1(b)) in contrast to normal control tissue (Figure 1(a)). IHC with iNOS antibody was observed on tissue sections of both control and cervical carcinoma epithelial cells. The iNOS was darkly stained in the cervical cancer tissue section, showing strong intensity with enhanced iNOS expression both in cytoplasm and nucleus (Figure 1(d)), and the cells show dark nuclei supporting the selective immunopositivity of iNOS, whereas the normal cervical epithelial cells were stained less (Figure 1(c)) showing weak iNOS expression.

### 3.2. Expression of iNOS in Cervical Tumor by RT-PCR

A real-time PCR experiment was performed to determine the expression of iNOS in cervical cancer tissue and confirmed the iNOS mRNA transcript. Our data revealed that iNOS expression was significantly upregulated and associated with cervical cancer tissue (Figure 1(e)) compared to that of control. Bar histogram clearly documented that cervical cancer patients showed a 12.21 ± 1.89-fold increase in iNOS expression compared to control patients (Figure 1(e)).

### 3.3. Validation of NOS2 Gene Relative Expression

**Figure 2:**
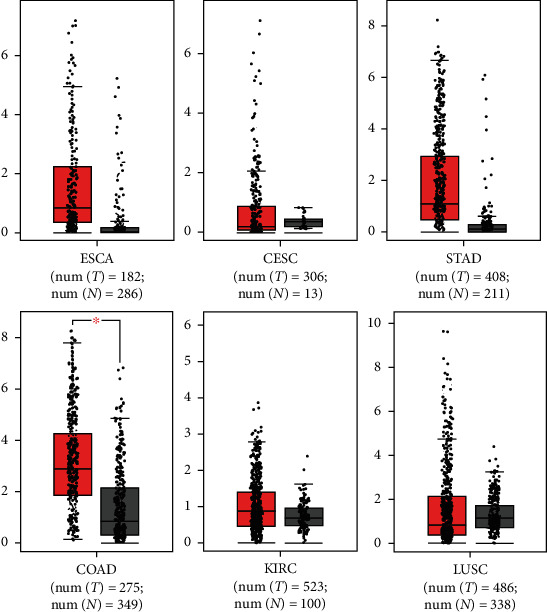
Differential expression of NOS2 gene: elevated NOS2 gene expression in different cancers. ESCA: esophageal carcinoma; CESC: cervical squamous cell carcinoma; STAD: stomach adenocarcinoma; COAD: colon adenocarcinoma; KIRC: kidney renal clear cell carcinoma; LUSC: lung squamous cell carcinoma.

To study the relative gene expression of NOS2 gene in several cancers, the Gene Expression Profiling Interactive Analysis (GEPIA), online software based on the TCGA, and Genotype-Tissue Expression (GTEx) datasets were used. The GEPIA-provided box plot tools for differential expression analysis of NOS2 in different cancers are illustrated in Figure 2.

### 3.4. Docking Study

**Figure 3:**
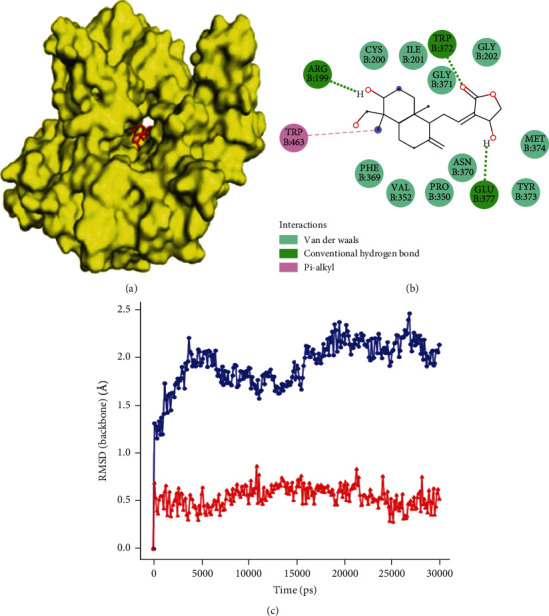
(a) 3D docking image of andrographolide with iNOS (nitric oxide acid), (b) two-dimensional representation of the interaction formed by andrographolide at the catalytical active site of iNOS, and (c) RMSD of native iNOS (4NOS) and protein-ligand complex (4NOS-andrographolide).

Molecular docking was applied to explore the mechanism of ligand binding activity and correlate with the ligand's binding score. The 3D structure of nitric oxide synthase protein was retrieved from the Protein Data Bank using the PDB ID: 4NOS (https://www.rcsb.org/structure/4nos). The docking of andrographolide and nitric oxide synthase was carried out through AutoDock Vina [30]. Inhibition constant Ki value for competitive inhibition denotes about one-half that of the IC_50_'s numerical value; if Ki value is higher, it affects the inhibitory activity of the enzyme. Based on the docking results and considering the Ki value, we selected the second-conformer since the inhibition constant value was found to be lower compared with the first conformer (Figure 3(a)). The binding energy of the protein-ligand complex was found to be -7.8 kcal/mol with inhibition constant (Ki) of 2.17 nM. Residues such as TRP372, GLU377, and ARG199 were found to have a strong binding affinity with the ligand during the docking (Figure 3(b)).  Table 2 shows the predicted binding energy values of the ligand with the target residues of iNOS (4NOS) during docking.

### 3.5. Molecular Dynamic Simulations

#### 3.5.1. RMSD

The molecular dynamic (MD) simulation was carried out to understand the interactions between andrographolide (ligand) and nitric oxide synthase (iNOS) in an aqueous environment. It is one of the most persuasive methods to study molecule physical nature and crucial properties, such as interaction, diffusion, and stability. A molecular dynamics was adopted to examine the protein and iNOS-andrographolide complex's structural dynamics and stability. In our present analysis, binding affinities of andrographolide and protein 4NOS were investigated by conducting static molecular docking. The binding affinity of iNOS-andrographolide was calculated using Schrodinger's Glide module, and it was found to be -7.8 kcal/mol. Molecular dynamic simulation of the protein complex was carried out for 30 ns after energy minimization using the OPLS force field [27]. Molecular dynamic trajectories were analyzed to identify interactions between andrographolide and protein. These molecular dynamic simulations were performed by Dell Precision T5810 Intel Xeon V3 processor with ten cores and twenty threads.

RMSD analysis revealed that the native state of the protein (iNOS) has more structural deviation with maximum fluctuations of ~2.4 Å. In contrast, in the complex form, iNOS-andrographolide complex fluctuations were reduced to a maximum of ~0.9 Å (Figure 3(c)). This reduction implies that the iNOS protein is structurally more stable upon binding to the ligand. Therefore, the ligand was found to be successfully stabilizing the iNOS (4NOS) [31].

#### 3.5.2. Torsional Degree of Freedom during MD Simulation Trajectory

**Figure 4:**
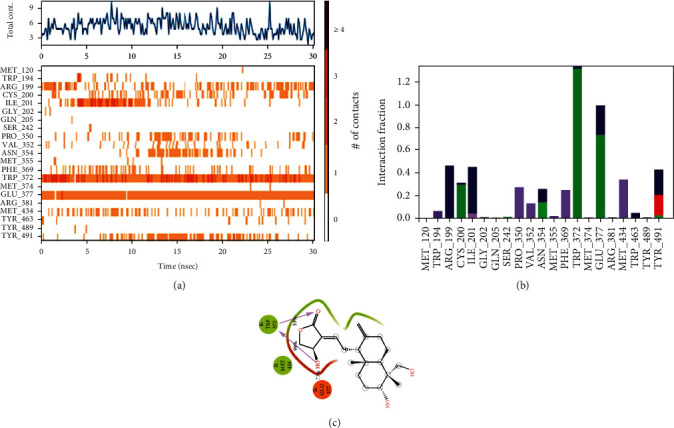
(a) Total contacts, (b) protein-ligand H bond, and (c) MD simulation hydrogen bond percentage.

Based on the contact timeline graph in Figure 4(a), interactions that occur more than 30% of the simulation time in the selected trajectory (30.12 ns) are shown where GLU377 and TRP372 residues are maintaining the contact throughout the simulation (Figures 4(a) and 4(b)) and the percentage involvement is 73% and 33%, respectively, and the same hydrogen bond interacting partners were observed in docking studies (Figure 4(c)) [32].

**Figure 5:**
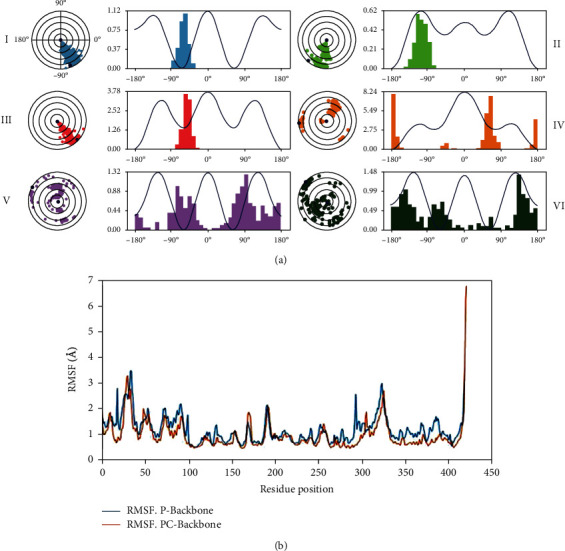
(a) Analysis of the torsional degree of freedom during MD simulation trajectory for the rotational bonds present in the andrographolide, (b) analysis of RMS fluctuation (RMSF) trajectories generated by Schrodinger (Desmond).

The ligand torsion dynamic graphs in Figure 5(a) summarize the formation of the rotational bond (RB) in the andrographolide (ligand) throughout the simulation trajectory (30.12 ns). The calculated torsional degree of freedom illustrated rotational bonds in the andrographolide. Andrographolide molecules hold six rotational bonds amid ligand positions (I) 17 to 23, (II) 14 to 15, (III) 12 to 14, (IV) 6 to 13, (V) 20 to 24, and (VI) 5 to 25. Bonds at positions (V) and (VI) were spinning 360° during the simulation; maximum torsion angles were observed with minimal potential energy, whereas (I) spin defined ranging from -35° to -90° with torsion angle maximum with minimal potential energy was observed (Figure 5(a)). Second rotational bond (II) -85° to -140° torsion angles were found to have maximum potential energy, and the third bond (III) -30° to -85° with average potential energy maximum torsion angles was observed. Finally, the fourth rotational bond (IV) was found with minimal potential energy torsion angles. The relation between torsion potentials and the rotational bond torsion angles revealed that confirmation of the ligand is stable and helps ligand in binding to protein effectively.

#### 3.5.3. P-RMSF

Residue contributing towards the native and complex protein structure can be assessed by the root mean square fluctuations (RMSFs) of each residue (Figure 5(b)). We inferred from the RMSF analysis that the iNOS protein in the native form has shown higher fluctuations than the iNOS-andrographolide complex with an RMSF distance of 0.45 Å for residues located between 260 and 400 residue positions (Figure 5(b)), while in the complex, the lowest peak was observed at the residue position 286. When compared with the residues, the total contacts with the ligand throughout the simulation as depicted in Figure 4(a), the corresponding residues were showing lower peaks and contributing to the stability of the protein. The overall average RMSF values of the native structure and complex were calculated as 1.165 Å and 0.96 Å, respectively (Figure 5(b)). Thus, RMSF analysis revealed that the lowest degree of flexibility was shown by the protein-ligand complex over the protein's length during the simulation (Figure 5(b)). Therefore, we concluded that these residues might play a role in directing the conformational transition. The values of RMSF of the iNOS protein and iNOS-andrographolide protein complex are illustrated in Table 3.

#### 3.5.4. Ligand Root Mean Fluctuation (L-RMSF)

The Ligand Root Mean Fluctuation (L-RMSF) of the ligand was performed on andrographolide to characterize the changes in the ligand atom's positions. The RMSF for an atom is calculated using formula:

(1) $$\begin{array}{c} \text{RMS}{\text{F}}_{i}=\sqrt{\frac{1}{T}\overset{T}{\underset{t=1}{\sum }}{\left(r_{i}^{1}\left(t\right)r_{i}\left(t_{\text{ref}}\right)\right)}^{2}}. \end{array}$$

**Figure 6:**
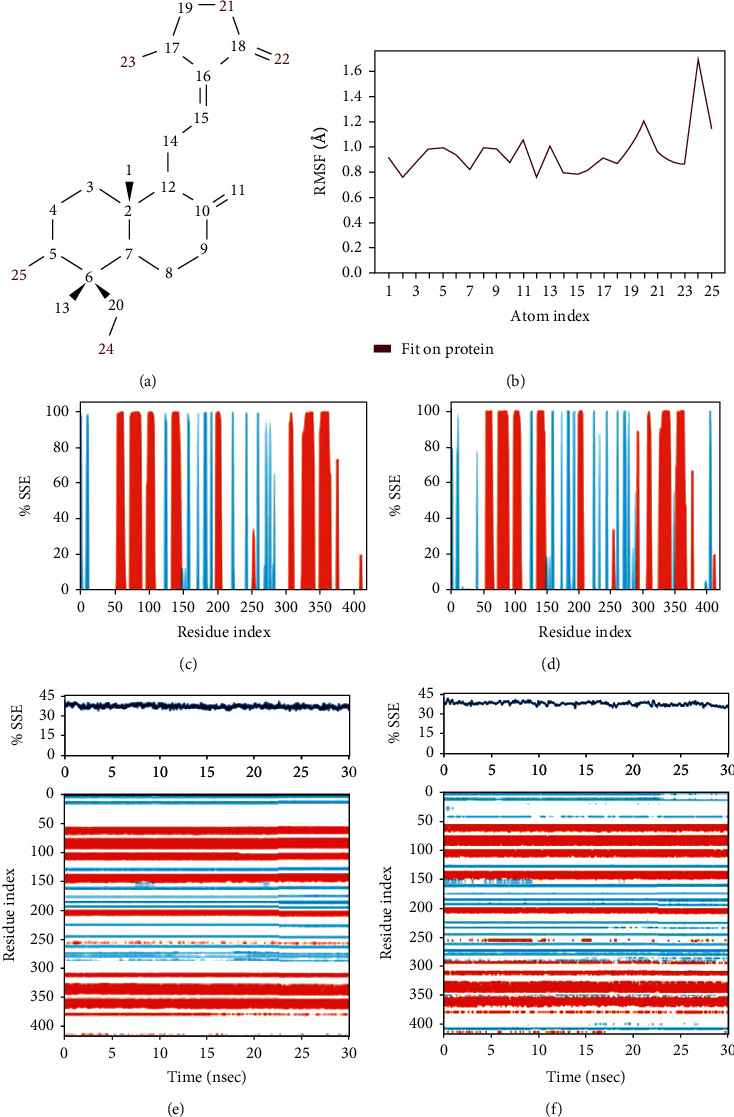
(a) 2D structure of andrographolide; (b) analysis of Ligand Root Mean Fluctuation (L-RMSF) of the andrographolide ligand. (c) SSE distributions by residue index throughout the protein structure iNOS. (d) SSE distribution by residue index of iNOS-andrographolide complex, summary of SSE composition for each trajectory frame throughout the simulation, (e) SSE composition for only protein, and (f) SSE composition for (protein complex).

Fluctuations of the andrographolide were broken down by atoms, corresponding to the 2D structure, and these atoms 20 (1.2 Å), 24 (1.7 Å), and 25 (1.13 Å) have demonstrated large fluctuations (Figures 6(a) and 6(b)). In contrast, the remaining atoms have minimal fluctuations well below 1 Å, indicating that the rest of the atoms were rooted inside the binding pocket. Atoms 20, 24, and 25 (Figures 6(a) and 6(b)) were solvent-exposed and could rotate easily around 6 to 13, 6 to 20, and 5 to 6, respectively. Further, we checked the drug-like properties of andrographolide, as shown in Table 4.

#### 3.5.5. SSE Distributions by Residue Index

To understand the protein stability in the native state and complex with the ligand, we have monitored the secondary structure element (SSE). We observed that in the protein native structure, alpha helix and beta strands cover 27.02% and 10.27%, respectively, constituted 37.3% of total residues remaining were loops, throughout the simulation (Figure 6(c)), whereas analysis revealed that the protein complex maintained about 27.06% helices and 12.77% strands constituted 40.59% of the residues leftover were loops (Figure 6(d)). Residues present at 50, loops (L) are converted to strands (S), 240, L to S, 300 L to helices (H) and S, and 400, L to S (Figures 6(e) and 6(f)). These SSE results substantiated that the protein has attained higher stability after forming the complex.

### 3.6. MTT Cytotoxicity Assay

**Figure 7:**
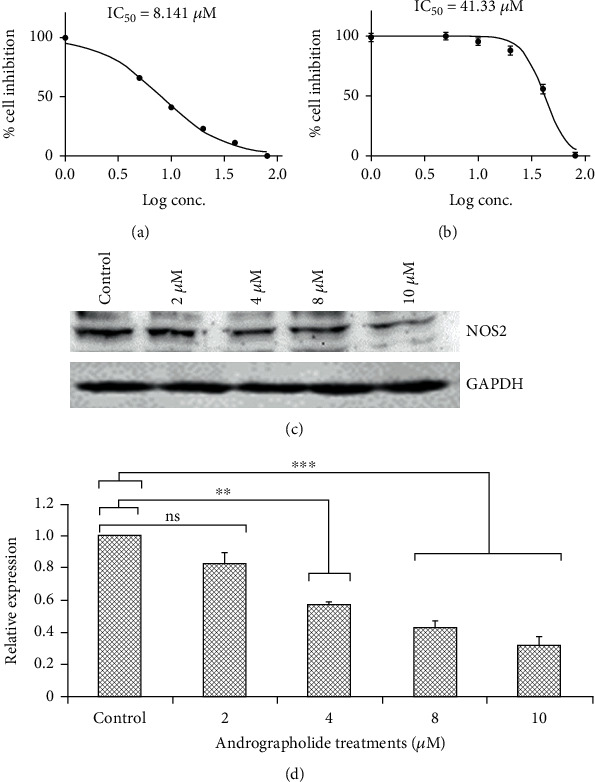
Cell viability due to andrographolide treatment was analyzed using MTT assay on (a) HeLa cells and (b) HEK cells; (c) Western blot analysis showing decrease in NOS2 expression in HeLa cells incubated with andrographolide, and GAPDH was used as control; (d) dose-dependent reduction of iNOS was observed in response to andrographolide treatments. ns: not significant. ∗∗ and ∗∗∗ indicate significance at *p* < 0.05 level.

The cytotoxicity and antiproliferative potentiality of different concentrations of andrographolide on HeLa and HEK cells (human embryonic kidney) were assessed using the MTT assay. The IC_50_ value after 24 h of andrographolide treatment was estimated to be 8.141 *μ*M/ml in HeLa cells (Figure 7(a)) and was less effective against HEK cells with 50% cell death at 41.33 *μ*M/ml (Figure 7(b)). It was observed that the IC_50_ value after 24 h in HeLa cells was 5-folds lower than that of normal cells. Andrographolide was found to be highly potent against cervical carcinoma HeLa cells in contrast to normal HEK cells and resulted in loss of cell viability in a concentration-dependent manner. Thus, andrographolide treatment was found to be cytotoxic against HeLa cells, and further, we aimed to focus further on anticancer assessment due to andrographolide.

### 3.7. Western Blot Analysis

Western blot analyses showed strong immunoreactivity towards antibodies of NOS2, and reduced NOS2 protein expression was observed in HeLa cells upon andrographolide treatment. We observed a dose-dependent decreased expression of NOS2 (Figures 7(c) and 7(d)), and the highest tested dose of andrographolide (10 *μ*M) was found to be most effective against HeLa cells. Data represents the consistency of three independent experiments with GAPDH as control.

### 3.8. DAPI (4′,6-Diamidino-2-phenylindole)

**Figure 8:**
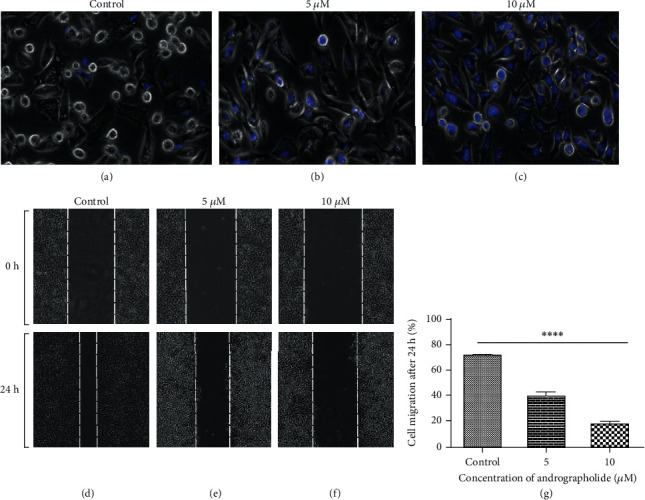
(a–c) DAPI-stained HeLa cell images of both (a) untreated control and treated andrographolide-induced apoptosis showing blue florescent nuclear fragmentation in HeLa cells at (b) 5 *μ*M and (c) 10 *μ*M, observed under an inverted fluorescence microscope (at magnification 20x). (d–g) Antimigration potency measured with andrographolide at (e) 5 *μ*M and (f) 10 *μ*M showing less migration than (d) control at 24 h. (g) Relative size of open scratch area (in percentage) in all the treatments and controls.

Cytotoxicity-induced apoptosis due to IC_50_ dose of andrographolide treatment on HeLa cells was observed using florescent DNA binding dye. The andrographolide-treated cervical HeLa cells showed characteristic apoptotic features containing nuclear fragmentation and irregular shape of the nucleus with intense blue florescence (Figures 8(b) and 8(c)). However, very less apoptotic cells having a round nucleus were observed in the case of untreated HeLa, showing faint or no blue dye validating alive cells (Figure 8(a)). The outcomes demonstrated that the andrographolide induces apoptosis in HeLa cells which corroborates with the previous reports.

### 3.9. *In Vitro* Wound Healing Scratch Assay

The wound healing scratch assay was used to determine the wound closure due to andrographolide treatment. To see the effect of andrographolide on migratory behavior of HeLa cells, the percentage of cell migration after 24 h was evaluated (Figures 8(d)–8(g)). Figure 8(d) illustrates the control with respect to all treatments. Results clearly revealed that andrographolide caused a significant inhibition of cell migration in a dose-dependent manner after 24 h treatments, with a wound area of 39.77 ± 2.54 (Figures 8(e) and 8(g)) and 17.96 ± 1.55 (Figures 8(f) and 8(g)) in 5 and 10 *μ*M, respectively, in contrast to the control, i.e., 71.59 ± 1.27 (Figure 8(d)).

### 3.10. Effect of Andrographolide on Cell Cycle

**Figure 9:**
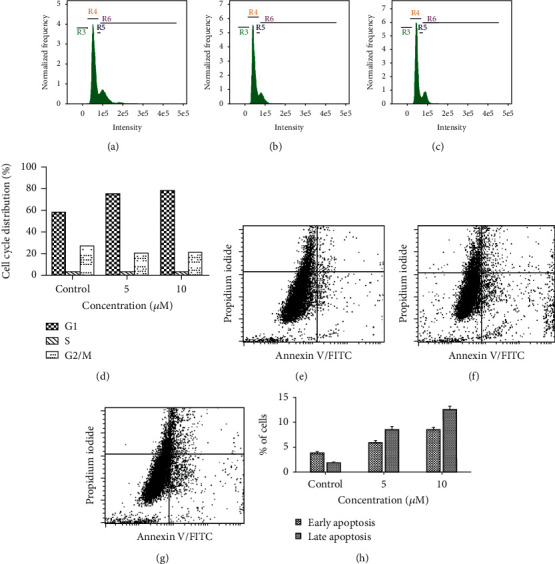
Flow cytometric analysis of andrographolide-treated HeLa cells showed dose-dependent (a–d) cell cycle arrest at G1/S transition phase, (e–h) Annexin V-FITC/PI-stained apoptotic cells. (a, e) Represent untreated control, (b, f) represent 5 *μ*M of andrographolide, and (c, g) represent 10 *μ*M of andrographolide. (d) Relative distribution of cell cycle percentage and (h) comparative percentage of the cell population in early and late apoptosis in control and andrographolide treatments.

Inhibition of cell cycle progression with the tested doses of andrographolide was evaluated; it exhibited dose-dependent elevation in the cell population in the G1 phase which was found to be 75.20% in 5 *μ*M (Figure 9(b)) and 78.40% in 10 *μ*M (Figure 9(c)), respectively, and in the untreated control, the cell population in the G1 phase was 59.40% (Figure 9(a)). The andrographolide treatment induced the cell cycle arrest at G1 to S transition phase (Figure 9(d)) in both the tested doses, and a notable decrease in cell population was observed in the G2/M phase in comparison to the untreated controls, and no notable changes were observed in the S phase due to induced cell cycle arrest at G1 to S transition phase (Figure 9(d)).

### 3.11. Andrographolide Induced Apoptosis

The effect of andrographolide on apoptosis was analyzed and evaluated in HeLa cells after 24 h treatment using Annexin V/PI dual staining. Annexin V/PI staining determined the percentage of apoptotic and nonapoptotic cells in both treated and untreated cells. A substantial increased uptake of Annexin V/PI with elevated cell count in both lower (Annexin V +/PI−) and upper right (Annexin V +/PI+) quadrants was seen in treated cells. Andrographolide treatment significantly induced early apoptosis; it was estimated to be 6.03% and 8.60% and late apoptosis as 8.74% and 12.71% in HeLa cells at 5 and 10 *μ*M/ml concentrations (Figures 9(f) and 9(g)). In contrast, the early and late apoptotic cell count (3.86% and 1.94%, respectively) was seen in untreated cells (Figure 9(e)). The viable cell (Annexin V−/PI−) population percentage was found to decrease in treated cells. The results confirmed that andrographolide significantly induced apoptosis-mediated cell death in HeLa cells in a concentration- or dose-dependent manner (Figure 9(h)).

## 4. Discussion

Andrographolide (diterpenoid lactone compound), an active and major constituent found in leaves of *Andrographis paniculata* [33], has been reported to demonstrate biological effects such as being an anti-inflammatory [34], antiviral [35], and anticancerous [36] agent and has also been reported as an immune booster [37]. Andrographolide extracts have also been found to inhibit iNOS expression [24]; it prevents the production of reactive oxygen species [22] and is known to protect against lipid peroxidation [38]. Additionally, antitumor property of andrographolide has been demonstrated in several cancer cell lines, including lung [39], hepatocellular carcinoma [40], breast [41], and colon [42] cancer cells. Andrographolide inhibits cancer cell proliferation by reducing cell viability [43] and arrests the cell cycle thereby inducing apoptosis [36, 44], and induced apoptosis could be targeted for treating cancer [45, 46]. Andrographolide exhibited anticancerous properties by inhibiting the cell proliferation, arresting cell cycle, and triggering apoptosis and cell death due to production of intracellular ROS [47, 48]. Depending on the types of cancer cells, the proapoptotic activity of andrographolide has been correlated with diverse mechanisms. Pratheeshkumar et al. [49] documented that apoptosis is induced due to andrographolide in melanoma B16F-10 cells, resulting in enhanced expression of Bax, caspase-3, and caspase-9 with downregulated Bcl-2. While in pancreatic cancer cells, apoptosis induced due to andrographolide treatment was due to STAT3 and Akt pathway activation [50]. Peng et al. [51] reported that andrographolide triggered the translocation of cytochrome c from the inner membrane of mitochondria to the cytosol leading to apoptosis. Protein binding residues of iNOS have been documented in previous literature [52, 53] and stated that the ligand andrographolide was found to have similar interactions with the active site residues of the protein. Thus, from the docking analysis, it is suggested that the ligand could bind to the target protein more consistently for maintaining the overall stability of the complex system. The results obtained from the current study are in agreement with the earlier reports published. Via flow cytometric analysis, we have determined that andrographolide induced dose-dependent cell growth inhibition by arresting cell cycle thus activating apoptosis.

Martínez-Estrada et al. [54] reported that andrographolide promoted Snail expression (EMT transcriptional repressor) which acts as a negative regulator of claudin-1 (repressing epithelial associated proteins) indicating that it is effective against migration mediated protein in cancer cells, thus establishing its potential antimigration and anti-invasive activities. Our results reconfirm that andrographolide exposure effectively inhibited HeLa cell proliferation and migration.

NO (the iNOS product) is an endogenous short lived signaling molecule found to be involved in inflammation, tumor formation, and metastasis [55]. NO acts alike free radicals and is highly reactive in biological systems, interacting with other free radicals or molecular oxygen to produce reactive oxygen species [56]. NO is known to obstruct cell proliferation and also leads the DNA damage by deamination, strand breakage, or DNA modification [56, 57] and impedes apoptosis [58]. Kumar and Kashyap [59] reported enhanced production of NO in breast cancer due to the elevated iNOS gene, responsible for damaging cellular components including DNA and proteins. The iNOS expression is reported in prostate cancer [60, 61], colon adenocarcinoma [62, 63], breast cancer [64, 65], melanoma cancer [66, 67], esophageal carcinoma [68], cervical squamous cell carcinoma [69], stomach adenocarcinoma [70], kidney renal clear cell carcinoma [71], and lung squamous cell carcinoma [72]. Our data from the clinical samples also revealed elevated levels of iNOS as found in most of the cancers across other ethnic population worldwide. An elevated iNOS level is a potential target for treatment in advanced stages of cancer. Cervical cancer is unarguably the most treatable cancer, wherein iNOS plays a crucial role in cancer advancement and can be effectively treated with andrographolide, a plant-based natural product being used as an adjuvant therapy along with the regular drugs and treatment regime.

Our findings *in vitro* have clearly shown that elevated iNOS expression in cervical cancer facilitates proliferation and migration and evades apoptosis. Andrographolide treatment to these cancer cells have shown to attenuate iNOS gene expression. iNOS expression is increased in cancer and stromal cells and exhibited increased vascularization, indicating that iNOS promoted angiogenesis in breast carcinoma thus showing correlation of metastasis with iNOS expression [64].

Further *in vivo* studies are warranted to provide insight and better understanding to our current outcomes. Several *in vivo* studies have demonstrated and confirmed the anti-inflammatory property of andrographolide, thereby depicting the significant reduction of iNOS in cancer tissues. Andrographolide administration stimulated the cytotoxicity and suppressed the tumor formation *in vivo* in oral carcinoma xenograft [73], HT-29 tumor xenografts, B16F0 melanoma [74], and colorectal carcinoma cells [75]. Zhang et al. [76] reported significant inhibition of tumor growth at both the early- and advanced-stages of insulinoma. Kumar et al. [41] reported that 20 *μ*M andrographolide treatment displayed significant inhibition of viability on both MDA-MB-231 and MCF-7 cells, also documenting that concentration of 50 to 100 *μ*M is nontoxic to normal cells. Therefore, literature authenticated that andrographolide treatment possesses the potentiality for inhibiting the tumor growth both *in vitro* and *in vivo* following different signaling pathways; thus, this study is aimed at foreseeing the effect of andrographolide on iNOS expression in cervical cancer HeLa cells, and the results are in accordance with the previous published reports.

From the results obtained, we establish the inhibitory effect of andrographolide on HeLa cells, and its property to specifically target and impede iNOS expression advocates its anticancerous property. An elevated iNOS level is an indicator of tumor aggressiveness and makes iNOS a compelling target for drug therapeutics. Andrographolide, a highly studied plant-derived natural product which is well known for its numerous beneficial properties and does not implicate any harmful side effect, can be introduced as an adjuvant drug in combination with the regular treatment regimen followed for the treatment of cervical cancer patients.

## 5. Conclusion

The current study has investigated the levels of iNOS in the cervical cancer patient samples, both at mRNA and protein levels. iNOS expression levels are found to be proportional to the stages of cancer and is highly elevated in the advanced stage of cancer. This formed the basis for the molecular docking, molecular dynamic, and simulation studies of iNOS protein with known ligands. Andrographolide presented encouraging unwavering results both in docking and simulation studies, hence selected for further *in vitro* analysis. Andrographolide efficiently and significantly inhibited the iNOS protein expression in a dose-dependent manner. Andrographolide also inhibited HeLa cell proliferation by arresting the cells at the G1-S phase, and further, it also induced cellular apoptosis as evinced from DAPI and Annexin V/PI staining. Our findings suggest that andrographolide exerts significant synergistic *in vitro* anticancer properties by impeding the ROS, and this paves way for their future application and use in cervical cancer treatment. The data mining has shown the significant association of the elevated levels of iNOS with an advanced stage of disease across various cancers; hence, andrographolide can also be used to treat various cancers and solid tumors. Further research is required to understand and explore the signaling mechanism being modulated by andrographolide in cervical carcinoma. Based on the current study. we strongly advocate andrographolide, a plant-derived natural product as a potent inhibitor of iNOS, with its antiproliferative and proapoptotic property as an alternative promising adjuvant drug in combination with the regular treatment regimen used in cervical cancer treatment. Andrographolide could facilitate delaying the cancer progression thereby increasing the life expectancy of the patient in an advanced stage of cancer and also will contribute towards improving the quality of the life of the affected women worldwide.

## Figures and Tables

**Table 1:** Primer sequences and amplicon sizes of iNOS and beta-actin genes used in the real-time qPCR.

Gene(s)	Forward primer	Reverse primer	Amplicon size (bp)
Beta-actin	CACCATTGGCAATGAGCGGTTC	AGGTCTTTGCGGATGTCCACGT	135
iNOS	GCTCTACACCTCCAATGTGACC	CTGCCGAGATTTGAGCCTCATG	136

**Table 2:** Top 5 conformers of predicted binding energy values of the protein-ligand complex during docking.

Conformers	Binding energy (kcal/mol)	Inhibition constant (Ki) (*μ*M)	Interacting residues
1	-8.40	697.83	ARG199, ASN370, TRP372, GLU377
2	-7.84	2.17	ARG199, TRP372, GLU377
3	-7.73	6.84	ARG199, TRP372, PRO 350, GLU377
4	-7.24	4.96	ARG199, VAL352, TRP372, GLU377
5	-6.23	26.91	ARG199, PHE369, TRP372, GLU377, TYR489

**Table 3:** Values of RMSF of the protein and protein complex.

Structures	PRO350	VAL352	MET355	ASN354	PHE369	TRP372	GLU377
Protein (iNOS)	0.95	1.27	1.04	1.11	1.30	1.07	1.01
Protein complex (iNOS-andrographolide)	0.57	0.57	0.57	0.65	0.75	0.79	0.58

**Table 4:** Drug-like properties of andrographolide.

Molecular weight (g/mol)	XLogP3	Hydrogen bond acceptor count	Hydrogen bond donor count	Index of refraction
350.4	2.2	5	3	1.568

## Data Availability

Data supporting the productivity of this investigation are available from the corresponding author upon request.
